# Complete genome sequence of two *Christensenella minuta* strains CIP 112228 and CIP 112229, isolated from human fecal samples

**DOI:** 10.1128/mra.00766-24

**Published:** 2024-10-29

**Authors:** Christiane Bouchier, Gérald Touak, Damien Rei, Dominique Clermont

**Affiliations:** 1 CIP, CRBIP, C2RT, Microbiology Department, Institut Pasteur, Paris, France; 2 NBX Biosciences, SAS, Centre ADVISOR, Montpellier, France; Wellesley College Department of Biological Sciences, Wellesley, Massachusetts, USA

**Keywords:** microbial genomics, human microbiome

## Abstract

*Christensenella minuta* is one of the representative bacterial species of the human gut microbiome. We report the complete genome sequence of two strains, *Christensenella minuta* CIP 112228 and CIP 112229, isolated from two healthy volunteers.

## ANNOUNCEMENT

In the microbiome-based therapy field, *Christensenella minuta* is reported as a potential probiotic for effective interventions on gut microbiomes and improvement of host health (1).

In order to increase the amount of genomic information on the species *C. minuta*, we report the complete genome sequence of two strains of *C. minuta* CIP 112228 and CIP 112229 from human feces belonging to the ancillary cohort “COSIMMGEN J” initiated for the study approved by the COMITE DE PROTECTION DES PERSONNES Ile de France 1 - DOSSIER : 2018-fév.-14819. These strains were isolated from two different healthy human stool samples, targeted by polyclonal antibodies against *Christensenella* spp. and sorted using flow cytometry under anaerobic conditions (2). They were grown in pre-reduced tryptone, peptone, glucose yeast extract medium (supplemented with 0.1% l-cysteine–HCl and vitamins) (3) at 37°C and under strict anaerobic conditions (5% H_2_/5% CO_2_/90% N_2_).

Genomic DNA used for Illumina and Nanopore sequencing were extracted from an 8 mL overnight culture via a Nanobind CBB kit (Pacific Biosciences, USA) following the manufacturer’s protocols. Illumina sequencing was performed by the Mutualized Platform for Microbiology (Institut Pasteur, Paris, France) following their standard workflow for library preparation (Nextera tagmentation kit, Illumina, USA) and an Illumina NextSeq 550 device using a 2 × 150 bp protocol. Paired-end reads were trimmed using fqCleanerER v23.12 workflow with a Phred quality score of 25 and read length ≥ 100 bases https://gitlab.pasteur.fr/GIPhy/fqCleanER. The same gDNA aliquots were also used for long-read sequencing using the ligation sequencing gDNA kit (SQK-NBD114.24, Oxford Nanopore Technologies, UK) and sequenced on a MinION Mk1C device for 48 h (Oxford Nanopore Technologies, UK) with R10.4 flow cell (FLO-MIN114, Oxford Nanopore Technologies, UK). The Dorado v0.3.0 tool was used to perform base calling. The ONT reads were processed using Filtlong v0.2.0 (length >5,000 bp) https://github.com/rrwick/Filtlong. The ONT-filtered reads were assembled with Flye v2.9 which generated a circularized contig for each strain (4). Error correction and polishing were then done using the ONT-filtered reads and Illumina-trimmed paired-end reads using Medaka v1.4.4 (Oxford Nanopore Technologies, UK). A second round of polishing was done using Illumina-trimmed reads with PolyPolish v0.5.0 (5). Rotation of the polished genome to start at the *dnaA* gene was carried out using the fixstart command in circlator v1.5.5 (6).

The completed genome of the strain CIP 112228 consists of one circular chromosome (2,770,534 bp, 51.87% GC) with an overall sequencing coverage of 71 × (ONT reads). The strain CIP 11229 has a circular genome size of 2,770,539 bp with a GC% of 51.87 and a genome coverage of 61 × (ONT reads).

The annotation of the genomes was performed using the NCBI Prokaryotic Genome Annotation Pipeline (version 6.7, March 2024) (Table 1).

**TABLE 1:** Genome features of the two *Christensenella minuta* strains

		Data for strain	
Feature		CIP112228	CIP112229
Long read	Number of reads	16 370	12 231
	Total bases	200 632 862	171 834 363
	Mean read length (bp)	13 811	14 049
	Mean read quality	17.6	17.6
	Median read length (bp)	10 220	10 296
	Median read quality	18.4	18.4
	N50 read length (bp)	15 802	16 535
Short read	Number of paired-end reads	2 142 245	6 160 111
	Read length (bp)	151	151
Assembly	Genome size (bp)	2 770 534	2 770 539
	Long-read coverage (×)	71	61
	Short-read coverage (×)	216	634
	GC content (%)	52	52
Annotation	Predicted number of coding sequences	2 539	2 539
	Number of rRNAs	6	6
	Number of tRNAs	51	51
	GenBank accession number	CP149434	CP149433

## Data Availability

The collection of volunteer’s samples has been reviewed by the COMITE DE PROTECTION DES PERSONNES Ile de France 1 - N°IRB / IORG # : IORG0009918 - N° ID-RCB : 0-A01353-36. The genome sequences were deposited at GenBank under the accession numbers CP149433 and CP149434. The Illumina paired-end reads can be found at SRA accession numbers SRR29733043 and SRR29733044. The ONT-filtered reads from the MinION MK1C run can be found at SRA accession numbers SRR29734392 and SRR29734393.

## References

[1] Ignatyeva O, Tolyneva D, Kovalyov A, Matkava L, Terekhov M, Kashtanova D, Zagainova A, Ivanov M, Yudin V, Makarov V, Keskinov A, Kraevoy S, Yudin S. 2023. Christensenella minuta, a new candidate next-generation probiotic: current evidence and future trajectories. Front Microbiol 14:1241259. doi:10.3389/fmicb.2023.1241259 38274765 PMC10808311

[2] Bellais S, Nehlich M, Ania M, Duquenoy A, Mazier W, van den Engh G, Baijer J, Treichel NS, Clavel T, Belotserkovsky I, Thomas V. 2022. Species-targeted sorting and cultivation of commensal bacteria from the gut microbiome using flow cytometry under anaerobic conditions. Microbiome 10:24. doi:10.1186/s40168-021-01206-7 35115054 PMC8812257

[3] Le Gratiet T, Poezevara T, Rouxel S, Houard E, Mazuet C, Chemaly M, Maréchal CL. 2020. Development of an innovative and quick method for the isolation of Clostridium botulinum strains involved in avian botulism outbreaks. Toxins (Basel) 12:42. doi:10.3390/toxins12010042 31936866 PMC7020472

[4] Kolmogorov M, Yuan J, Lin Y, Pevzner PA. 2019. Assembly of long, error-prone reads using repeat graphs. Nat Biotechnol 37:540–546. doi:10.1038/s41587-019-0072-8 30936562

[5] Wick RR, Holt KE. 2022. Polypolish: short-read polishing of long-read bacterial genome assemblies. PLoS Comput Biol 18:e1009802. doi:10.1371/journal.pcbi.1009802 35073327 PMC8812927

[6] Hunt M, Silva ND, Otto TD, Parkhill J, Keane JA, Harris SR. 2015. Circlator: automated circularization of genome assemblies using long sequencing reads. Genome Biol 16:294. doi:10.1186/s13059-015-0849-0 26714481 PMC4699355

